# Mini-review of interesting properties in Mn_2_CoAl bulk and films

**DOI:** 10.3389/fchem.2022.1054337

**Published:** 2022-10-19

**Authors:** Ying Yang

**Affiliations:** College of Physics and Electronic Engineering, Chongqing Normal University, Chongqing, China

**Keywords:** Mn_2_CoAl, spin-gapless materials, Heusler, DFT, density functional theory, film, substrate system

## Abstract

Heusler compounds exhibit many interesting properties, such as high thermopower, magnetocaloric properties, and even topological insulator states. Heusler Mn_2_CoAl alloy has been experimentally and theoretically proposed as a promising spin-gapless semiconductor with novel electronic, magnetic, spintronic, transport, and topological properties. Furthermore, the spin-gapless semiconducting-like behaviors are also predicted in Mn_2_CoAl films by measuring the transport and magnetic properties. This mini-review systematically summarizes the interesting properties of Mn_2_CoAl bulk and Mn_2_CoAl-based films. This mini-review is hoped to guide further experimental investigations and applications in the particular scientific community.

## Introduction

On the one side, in 2008, Liu et al. (2008) investigated the structural, electronic, and magnetic properties of Mn_2_CoZ (*Z* = Al, Si, Ge, Sn, and Sb) alloys with Hg_2_CuTi-type structure with the help of first-principles calculations, and they stated that all the Mn_2_CoZ alloys belong to ferrimagnetic half-metals (Fang et al., 2002; Dowben and Skomski, 2004; Müller et al., 2009; Ashton et al., 2017). It is noteworthy that the spin-gapless semiconducting state of Mn_2_CoAl has not been mentioned by Liu et al. (2008). Moreover, Liu et al. (2008) stated that the Mn_2_CoZ (Z = Al, Si, Ge, Sn, and Sb) alloys follow the Slater-Pauling rule M_t_ =N_V_−24 (M_t_ denotes the total magnetic moment and the N_V_ is the valence electrons in both spin channels). Then, Liu et al. (2008) successfully synthesized the Hg_2_CuTi-type Mn_2_CoZ alloys, in which the two Mn atoms exhibit different magnetic behaviors. In the same year, Xing et al. (2008) also proposed the half-metallic ferrimagnetism of the Heusler alloys Mn_2_CoZ (Z = Al, Ga, Si, Ge) using the first-principles plane-wave pseudopotential method. Moreover, they (Xing et al., 2008) pointed out that the half-metallic states in Mn_2_CoZ can maintain in a large range of lattice constants, reflecting robust half-metallic behaviors. In 2011, Meinert, Schmalhorst, and Reiss (Meinert et al., 2011) studied the complex magnetic interactions between the constituents and the Curie temperatures using first-principles calculations. Surprisingly, the Curie temperatures of Mn_2_CoAl/Ga/In are all above 800 K.

On the other side, in 2008, Wang, (2008) proposed the concept of a spin-gapless semiconductor. The spin-gapless semiconductor is a new class of zero-gap materials. Spin-gapless semiconductors for practical use should feature 1) completely spin polarized carriers; 2) high mobility of carriers; 3) zero or negligibly small excitation energy of electrons from the valence to the conduction band; 4) easy switching between electron and hole modes by tuning the Fermi level, owing to the ambipolar nature of the band gap (For type II spin-gapless semiconductors). Wang, (2008) proposed that by introducing magnetic ions into the parent nonmagnetic gapless compounds, such as PbPdO_2_, the spin-gapless semiconducting state can appear. Based on his work, a series of spin-gapless semiconductors (Li et al., 2009; Gao et al., 2015; Wang et al., 2016a; Galanakis et al., 2016; Gao et al., 2016; He et al., 2017; Wang, 2017; Deng et al., 2018a; Huang et al., 2019a; Nadeem et al., 2020; Yue et al., 2020; Yang et al., 2021) with parabolic or linear dispersion between energy and momentum are proposed. More interestingly, topological signatures, such as Dirac point and nodal line states, can be found in spin-gapless semiconductors. For example, He et al. (2017) proposed that NiCl_3_ monolayer is a near-room-temperature Dirac spin-gapless semiconductor when the spin-orbital coupling (SOC) is absent. When SOC is added, it becomes an intrinsic Chern insulator with a large non-trivial band gap of ∼24 meV, at which the quantum anomalous Hall effect could be observed. In 2018, Zhang et al. (2018) proposed the nodal ring spin-gapless semiconducting state in a 2D HK lattice *via* first-principle calculations. In 2022, Ding et al. (2022) summarized almost all the predicted nodal ring/line spin-gapless semiconductors in 2D and 3D materials (Guan et al., 2013; Li and Yang, 2013; Ding and Wang, 2015; Wang et al., 2016b; Rasool et al., 2016; Wang et al., 2017c; Liu et al., 2017; Deng et al., 2018b; Wang et al., 2018; Huang et al., 2019b; Wu et al., 2020b; Guo et al., 2020; Li et al., 2021; Wang et al., 2021; Ji et al., 2022; Wu et al., 2022) in the past 3 years. Remarkably, they (Ding et al., 2022) also provided three valuable suggestions for the future theoretical design of nodal ring/line spin-gapless semiconductors. Moreover, some quaternary Heusler SGSs (Bainsla et al., 2015; Rani et al., 2019) have been prepared, and their interesting gapless behavior has been confirmed. For the verification of the spin-gapless semiconducting property, their specific transport behavior has been widely measured and accepted as strong evidence.

In 2013, the spin-gapless semiconducting state in Mn_2_CoAl has been experimentally confirmed by Ouardi et al. (2013a) according to the transport behaviors. They reported that Mn_2_CoAl with robust spin polarization is a promising material for room-temperature semiconductor spintronics. In this mini-review, the properties of Mn_2_CoAl bulk, Mn_2_CoAl [001] surface, Mn_2_CoAl/GaAs heterostructures, Mn_2_CoAl/Ag/Mn_2_CoAl current-perpendicular-to-plane spin valves, MgO/Mn_2_CoAl/Pd trilayers, and various types of Mn_2_CoAl films are reviewed in details. This mini-review aims to provide an improved understanding of the properties for Mn_2_CoAl that have been reported in the last 14 years.

## Structural, electronic, elastic, and thermodynamic properties for Mn_2_CoAl bulk

**FIGURE 1:**
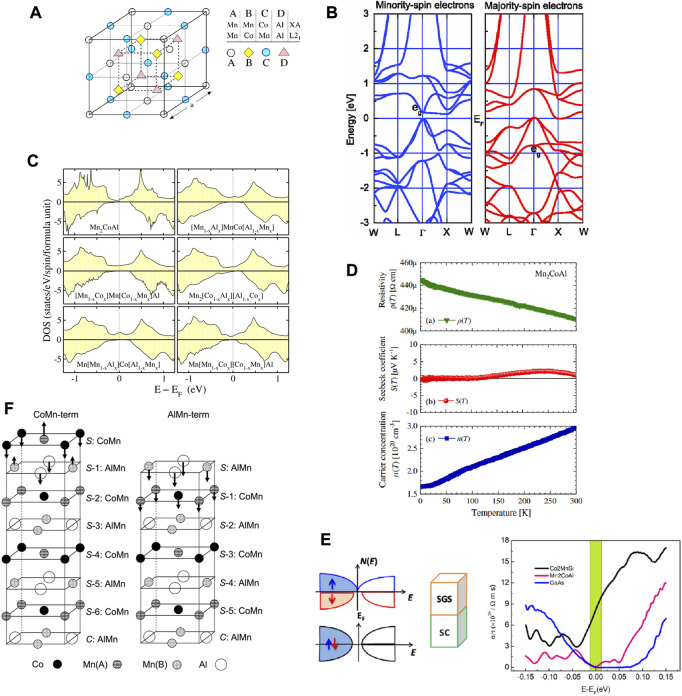
**(A)** Crystal structures of XA and L2_1_ type Mn_2_CoAl. **(B)** Band structures of XA Mn_2_CoAl. **(C)** The density of states of Mn_2_CoAl by considering atomic swaps. **(D)** Measured resistivity, S(T), and n for Mn_2_CoAl sample under different temperatures. **(E)** Schematic diagrams of spin-gapless semiconductor (SGS)/semiconductor (SC) spin injection scheme and the calculated room temperature conductivities for half-metal Co2MnSi, spin-gapless semiconductor Mn_2_CoAl, and semiconductor GaAs. **(F)** The (001) Mn_2_CoAl surface with Co-Mn and Al-Mn terminated surfaces. Reproduced from Refs. (Ouardi et al., 2013a; Li and Jin, 2013; Galanakis et al., 2014; Xu et al., 2019) with permissions.

The crystal structure of the Heusler alloys can be viewed as a cubic structure with four interpenetrating f.c.c. Sublattices A, B, C, D (see Figure 1A). Normally, full-Heusler alloys X_2_YZ can host two types of structures (XA and L2_1_ type structures). As shown in Figure 1A, we exhibited the XA type and L2_1_ type Mn_2_CoAl. MnMnCoAl and MnCoMnAl represent the XA type Mn_2_CoAl and L2_1_ type Mn_2_CoAl, respectively. As mentioned above, Liu et al. (2008) have determined that the crystal structure of Heusler Mn_2_CoAl should be XA type (i.e., Hg_2_CuTi) instead of L2_1_. We would like to point out that other researchers have widely investigated the competition between XA and L2_1_ atomic ordering in full Heusler alloys (Wang et al., 2017a; Wang et al., 2017b; Han et al., 2019a; Han et al., 2019b; Wu et al., 2019; Wu et al., 2020a) in recent years.

Based on the determined XA structure, the spin-polarized band structures for Mn_2_CoAl are exhibited in Figure 1B. Obviously, in the minority-spin channel, a direct band gap can be found around the Fermi level, whereas, in the majority-spin channel, an indirect zero-band gap can be observed. Note that, such band structures allow for tunable spin transport. Mn_2_CoAl hosts a high Curie temperature of ∼720 K and a total M_t_ of 2 μ_B_. In 2015, Chen et al. (2015) investigated the spin-gapless semiconducting states and the dynamical stability of Mn_2_CoAl, and they found that the spin-gapless semiconducting states and the dynamical stability can maintain with a pressure less than 25 GPa. The calculated elastic modulus, shear modulus, Yong’s modulus, and Pugh’s ration for Mn_2_CoAl under equilibrium lattice constants are 185.778, 77.782, 204.768 GPa, and 2.39, respectively. Moreover, with the help of the quasiharmonic Debye model, the pressure and temperature dependences of normalized volume V/V_0_, bulk modulus B, thermal expansivity, Gruneisen parameter, heat capacity, and Debye temperature are evaluated by Chen et al. (2015) for Mn_2_CoAl up to 25 GPa. The above-listed data can be viewed as a reference for the follow-up experimental studies.

## Conditions for spin-gapless semiconducting behavior in Mn_2_CoAl bulk

In 2014, Galanakis et al. (2014) examined the conditions for the spin-gapless semiconducting state in Mn_2_CoAl *via* first-principle calculations. They (Galanakis et al., 2014) showed that the spin-gapless semiconducting states in Mn_2_CoAl can be kept by applying the tetragonalization of the lattice. However, the spin-gapless semiconducting states in Mn_2_CoAl are disappeared by considering the atomic swaps. Swapping the atoms induces a physics nature transition from a spin-gapless semiconducting state to a half-metallic state (as shown in Figure 1C). Furthermore, they (Galanakis et al., 2014) also pointed out that the appearance of Co-surplus will lead to half-metallic states.

## Experimentally verified for the spin-gapless semiconducting behavior in Mn_2_CoAl bulk

In 2013, the transport measurements of Mn_2_CoAl bulk were performed by Ouardi *et al.* (Ouardi et al., 2013b; Ouardi et al., 2019) *via* a physical properties measurement system. They pointed out that Mn_2_CoAl bulk hosts a nonmetallic resistivity (see Figure 1D). Note that the resistivity of Mn_2_CoAl bulk is two orders of magnitude higher than that of Co_2_FeSi metal. From Figure 1D, one finds that the carrier concentration (*n*) is temperature independent, and the Seebeck coefficient (*S(T)*) is vanishing. The reason for the appearance of a vanishing *S(T)* is the compensation of the electron and hole. Note that the temperature-independent *n* is a main feature for the gapless systems.

Combining the transport and magnetic properties, one can conclude that Mn_2_CoAl bulk should be a spin-gapless semiconductor. Ouardi *et al.* (Ouardi et al., 2013b; Ouardi et al., 2019) also reported the magnetoresistance results and the anomalous Hall conductivity of Mn_2_CoAl bulk, suggesting Mn_2_CoAl is a novel spintronic material.

Note that the transport and magnetic properties cannot be viewed as the only criteria to judge the spin-gapless semiconducting states in Heusler alloys. The microstructure observations (Xu et al., 2019) should also be considered to validate the spin-gapless semiconducting states in Heusler alloys.

## New spin injection scheme based on Mn_2_CoAl

Spin injection efficiency based on conventional and/or half-metallic ferromagnets/semiconductors is greatly limited by the Schmidt barrier due to conductivity mismatch. In 2015, Xu et al. (2019) proposed that the spin-gapless semiconductor, such as Mn_2_CoAl, can be used to replace the conventional and/or half-metallic ferromagnets and form a spin-gapless semiconductor/semiconductor heterostructure (as shown in Figure 1E).

From Figure 1E, we listed the calculated room temperature conductivities for half-metal Co_2_MnSi, spin-gapless semiconductor Mn_2_CoAl, and semiconductor GaAs. The figure shows that the conductivity of spin-gapless semiconductor Mn_2_CoAl is much lower than that of half-metal Co_2_MnSi. More importantly, the conductivity of spin-gapless semiconductor Mn_2_CoAl is very close to that of semiconductor GaAs. Hence, using spin-gapless semiconductors as the magnetic injectors can reduce the conductive mismatch and enhance the spin injection efficiency.

We would like to point out that the thermodynamic stability, magnetism, and half metallicity of Mn_2_CoAl/GaAs (0 0 1) interface have been studied by Feng *et al.* (Feng et al., 2015a) *via* first principle calculations in 2015. In this same year, Feng *et al.* (Feng et al., 2015b) studied the effect of disorder on the electronic and magnetic properties of Mn_2_CoAl/GaAs heterostructures from theory.

## Electronic structures, magnetism, and half-metallicity for [001] Mn_2_CoAl surface

In 2013, Li and Jin (2013) studied the electronic, magnetic, and half-metallic properties of the Mn_2_CoAl [001] surface by first-principles calculation. As shown in Figure 1F, two types of surface terminations, i.e., AlMn terminated and CoMn terminated surfaces, are considered by Li and Jin (2013). They Li and Jin (2013) reported that the AlMn-terminated Mn_2_CoAl surface hosts half-metallic behavior, whereas the CoMn-terminated Mn_2_CoAl surface does not have the half-metallic behavior.

In 2018, Meng *et al.* (Wei et al., 2018) investigated the interfacial electronic, magnetic, and spin transport properties of Mn_2_CoAl/Ag/Mn_2_CoAl current-perpendicular-to-plane spin valves. Interestingly, they (Wei et al., 2018) pointed out that the MnCo^T^-terminated interface enjoys the largest interface spin polarization of 78% and magnetoresistance ratio of 2,886%.

## Experimental Mn_2_CoAl based films

**FIGURE 2:**
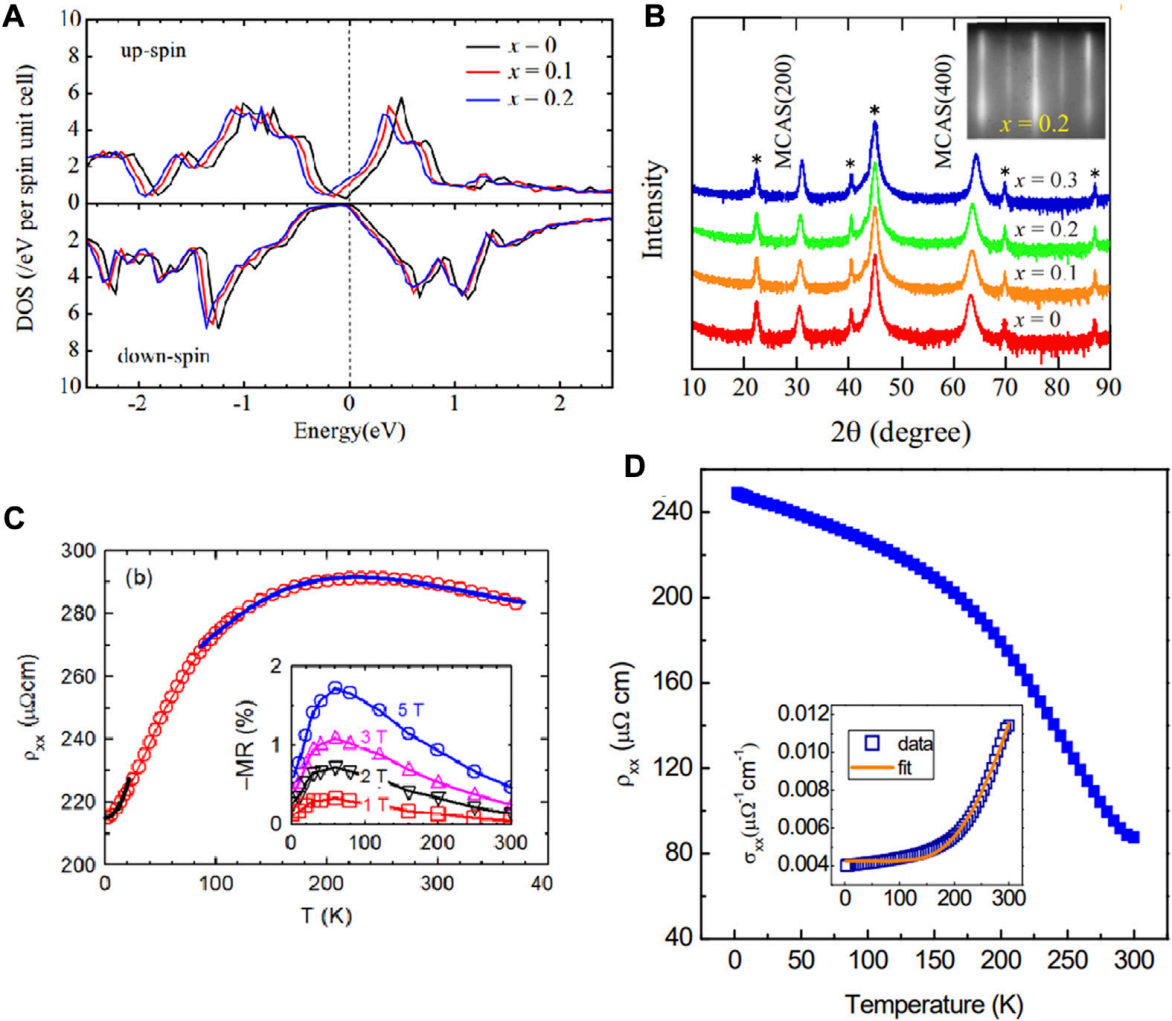
**(A)** Density of states for Mn_2_CoAl_1-x_Si_x_ (x = 0, 0.1, and 0.2). **(B)** The θ-2θ x-ray diffraction of the Mn_2_CoAl_1-x_Si_x_ films. **(C)** Resistivity *versus* temperature (red circles). Insert shows magnetoresistance at several magnetic fields. **(D)** The relationship between the resistivity and temperature was measured at zero magnetic fields. The inset shows the fitting of conductivity. Reproduced from Refs. (Jamer et al., 2013; Xu et al., 2014; Arima et al., 2018) with permissions.

To this date, some investigations are performed on Mn_2_CoAl-based films in the experiment. Hence, this section reviews some interesting properties focusing on the Mn_2_CoAl-based films. In 2018, Arima et al. (2018) studied the electronic structures and the anomalous Hall conductivity of Si-substituted Mn_2_CoAl epitaxial films. Based on the calculated density of states in Figure 2A, one finds that the spin-gapless semiconducting-like behavior can be maintained in Mn_2_CoAl_1-x_Si_x_ with x < 0.2. Mn_2_CoAl_1-x_Si_x_ films (0 ≤ x ≤ 0.3) were grown on MgAl_2_O_4_ (100) substrates by molecular beam epitaxy. We collect the θ-2θ x-ray diffraction of the Mn_2_CoAl_1-x_Si_x_ films in Figure 2B. Arima et al. (2018) stated that the electrical conductivity of Mn_2_CoAl_0.8_Si_0.2_ film is 2340 S/cm, which is closes to that of Mn_2_CoAl bulk (2440 S/cm).

In 2013, Jamer et al. (2013) prepared Mn_2_CoAl films on GaAs (001) substrates using molecular beam epitaxy. From Figure 2C, one finds the low-temperature resistivity of a 69 nm thick Mn_2_CoAl film is about 220 μΩcm, and a metallic-like behavior at low temperatures. They (Jamer et al., 2014) also reported that the Mn_2_CoAl films on GaAs (001) substrates exhibited varying amounts of disorder under different annealing temperatures, resulting in the magnetism changing. As the annealing temperature increases, the M_t_ increases.

In 2014, Xu et al. (2014) prepared Mn_2_CoAl films on the thermally oxidized Si substrates by magnetron sputtering deposition. They found that the films host a semiconducting-like resistivity and linear magnetoresistance in the whole region (see Figure 2D). Xu et al. (2014) also reported the unusually low anomalous Hall conductivity, saturation magnetization (1.94 μ_B_ at 5 K), and the Curie temperature (∼550 K). Usually, the results mentioned above are the transport signatures of spin-gapless semiconductors. In 2018, Chen et al. (2018) prepared Mn_2_CoAl films on MgO (001) substrates using molecular beam epitaxy. Their electro- and magneto-transport results showed that the Mn_2_CoAl hosts spin gapless semiconducting mechanisms at low temperatures. In 2017, Ludbrook and the collaborators (Ludbrook et al., 2017) showed that the MgO/Mn_2_CoAl/Pd trilayers could exhibit a novel topological Hall effect in temperatures between 3 K and 280 K. The topological Hall effect is evidence of skyrmions.

## Summary

In this mini-review, the electronic, magnetic, and transport properties of Mn_2_CoAl bulk, Mn_2_CoAl [001] surface, Mn_2_CoAl/GaAs heterostructures, Mn_2_CoAl/Ag/Mn_2_CoAl current-perpendicular-to-plane spin valves, MgO/Mn_2_CoAl/Pd trilayers, and various types of Mn_2_CoAl films (with MgAl_2_O_4_, GaAs, MgO, and thermally oxidized Si substrates) are reviewed in details. A new spin injection scheme based on Mn_2_CoAl and normal semiconductors is also summarized in this mini-review.
